# Discovery of the Largest Orbweaving Spider Species: The Evolution of Gigantism in *Nephila*

**DOI:** 10.1371/journal.pone.0007516

**Published:** 2009-10-21

**Authors:** Matjaž Kuntner, Jonathan A. Coddington

**Affiliations:** 1 Institute of Biology, Scientific Research Centre of the Slovenian Academy of Sciences and Arts, Ljubljana, Slovenia; 2 Department of Entomology, National Museum of Natural History, Smithsonian Institution, Washington D. C., United States of America; Landcare Research, New Zealand

## Abstract

**Background:**

More than 41,000 spider species are known with about 400–500 added each year, but for some well-known groups, such as the giant golden orbweavers, *Nephila*, the last valid described species dates from the 19^th^ century. *Nephila* are renowned for being the largest web-spinning spiders, making the largest orb webs, and are model organisms for the study of extreme sexual size dimorphism (SSD) and sexual biology. Here, we report on the discovery of a new, giant *Nephila* species from Africa and Madagascar, and review size evolution and SSD in Nephilidae.

**Methodology:**

We formally describe *N. komaci* sp. nov., the largest web spinning species known, and place the species in phylogenetic context to reconstruct the evolution of mean size (via squared change parsimony). We then test female and male mean size correlation using phylogenetically independent contrasts, and simulate nephilid body size evolution using Monte Carlo statistics.

**Conclusions:**

*Nephila* females increased in size almost monotonically to establish a mostly African clade of true giants. In contrast, *Nephila* male size is effectively decoupled and hovers around values roughly one fifth of female size. Although *N. komaci* females are the largest *Nephila* yet discovered, the males are also large and thus their SSD is not exceptional.

## Introduction

**Figure 1:**
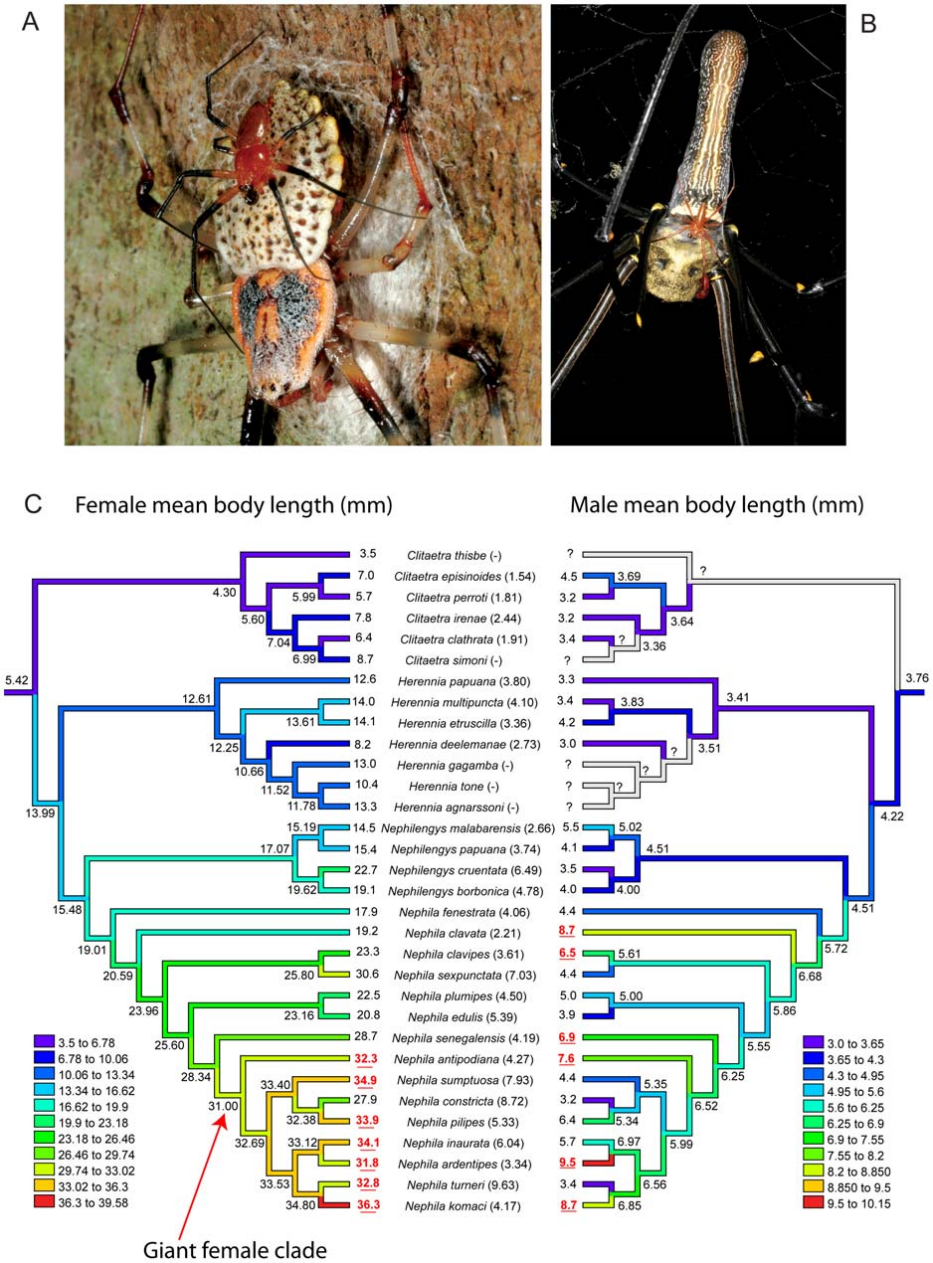
Moderate and extreme sexual size dimorphism and the evolution of body size in nephilid spiders. A, Moderate SSD - male resting on female (*Herennia multipuncta*). B, Extreme SSD - male walking over female (*Nephila pilipes*). C, Female mean body size increases monotonically sevenfold, but male size oscillates within a threefold range (SSD in parentheses; we arbitrarily define extreme SSD with females more than five times male size). Red underlined values significantly exceed Monte Carlo simulated size ranges. Gray denotes unknown males. Female and male size evolution are independent (n = 26; r^2^ = 0.055; *p = 0.787*).

The origin and maintenance of sexual size dimorphism (SSD) are much debated topics in evolutionary biology [1], [2], [3]. Spiders in general [4], [5], [6], [7], [8], and the orbweaving family Nephilidae in particular (e.g. *Herennia*, Fig. 1A, and especially *Nephila*, Fig. 1B) are becoming model organisms for the studies of extreme, female-biased SSD and its consequences for sexual biology [9], [10], [11], [12], [13], [14], [15]. Previous studies have focused on the relative importance of selection for large female size versus selection for small male size [16] and the current phylogenetic evidence suggests that extreme SSD in orbweaving spiders, nephilids included, is almost always due to female gigantism rather than male dwarfism [5], [16], [17], [18], [19]. However, prior studies all focused on individual species or on supraspecific phylogenetic levels. Combined with the new species described here, a recent species level nephilid phylogeny [20] makes possible the most detailed analysis of size change in nephilids to date, and thus should enable more rigorous hypotheses about selective forces affecting SSD in spiders.

*Nephila* contains the largest web-spinning spiders (∼10 cm leg span), which make the largest orb webs (>1 m diam.) [20], [21]. Out of 150 available scientific names, only 15 *Nephila* species are valid [22]. Linnaeus described the first *Nephila* species in 1767 (now *N. clavipes*) and Karsch described the last genuinely new *Nephila* in 1879 (*N. constricta*); all more recent descriptions are synonyms. This paper reports the discovery of the first new *Nephila* species since 1879. The first specimen, a huge, distinctly different female collected in 1978 at Sodwana Bay, South Africa, was discovered in 2000 in the collections from Pretoria. Two expeditions specifically to find this species were unsuccessful, suggesting that perhaps the form was a hybrid or extinct. Then in 2003 a second, unmistakably conspecific specimen from Madagascar was discovered in a Viennese museum, thus weakening the hybrid hypothesis. Failure to find additional specimens in more than 2500 samples from 37 museums seemed to support the extinction hypothesis. However, two additional females and a male were recently collected in Tembe Elephant Park by South African colleagues, and it is now clear that *N. komaci* is a valid, new extant *Nephila* species.

Here, we provide a formal description of *Nephila komaci* sp. nov., add it to the existing nephilid phylogenetic matrix [20], reconstruct the evolution of mean female and male size, and test their correlation using phylogenetically independent contrasts.

## Results and Discussion

The genus *Nephila* already contained the largest orbweaving spiders, but *N. komaci* now becomes the largest *Nephila* species known (Fig. 1C). Our phylogeny shows that nephilid female size increases monotonically (binomial test of ancestral *Nephila* nodes leading to *N. komaci*, n = 8, *p = 0.004*) and roughly sevenfold from implied ancestral values (Fig. 1C). This evolutionary trend is mainly due to *Nephila*; it alone is significantly larger than the family average or compared to any combination of the remaining genera (t test, n = 31, *p = 0.017*). The largest *Nephila* species all belong to one “giant female” clade, containing African species (e.g. *N. komaci*) and the Australasian *N. antipodiana* and *N. pilipes* (Fig. 1B). Throughout the family, females significantly more often increase in size rather than decrease at speciation events (binomial test of all paired ancestor-descendant nodes, n = 62, *p = 0.049*). Monte Carlo simulation shows that the “giant female” clade, except *N. constricta*, significantly exceeds expected body size (Fig. 1C, n = 15,000 replicates, p<0.05). However, nephilid male size oscillates within a threefold range (Fig. 1C), shows no significant trend with phylogeny, and is decoupled and independent from the evolution of female size (n = 26, r^2^ = 0.055, *p = 0.787*). Monte Carlo simulation of male size, however, shows that males sporadically achieve significantly large sizes (Fig. 1C).

**Figure 2:**
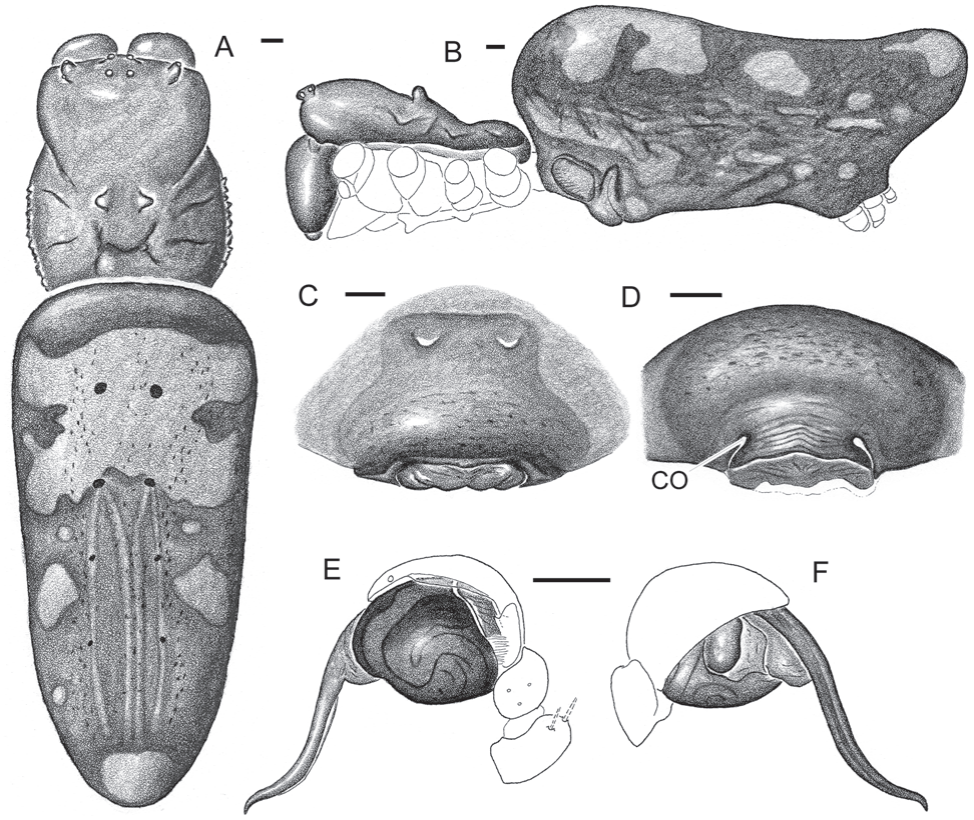
*Nephila komaci* sp. nov. A–D, Female paratype (from Sodwana Bay, South Africa). A, habitus (legs omitted), dorsal. B, same, lateral. C–D, external epigynal morphology. C, ventral. D, posterior. E–F, male palp (from Zanzibar). E, ectal. F, mesal. Scale bars A–B = 1.0 mm, C–F = 0.5 mm. CO  =  copulatory opening.

These species-level data reinforce *Nephila* sexual size dimorphism as female gigantism [5], [16], rather than male dwarfism [18], [19]. Large *Nephila* females may experience less predation [17] and, apparently at thresholds of roughly 28 mm body length, are freed to respond dramatically to fecundity selection for large size [17], [23]. First male advantage, sperm competition, or climbing ability favor small size via early maturation, but direct male-male competition and female cannibalism of males favor large size [7], [9], [24], [25]. Significant deviations from expected male size are all increases, suggesting that males do track females to some extent, but these increases are phylogenetically scattered (Fig. 1C). As a new member of the distal (giant) *Nephila* clade, *N. komaci* should be at the forefront of nephilid sexual size dimorphism research. If any other viable populations of this distinctive species exist they ought to be easy to locate (Fig. 2). Although the distribution data are currently scarce, the species may be threatened or endangered. It is nowhere abundant, the range is apparently restricted, and all known localities lie within two endangered biodiversity hotspots: Maputaland-Pondoland-Albany and Madagascar.

Taxonomy

Nephilidae Simon 1894 *sensu* Kuntner [26]

Nephilinae Simon 1894 *sensu* Kuntner [26]

*Nephila* Leach 1815

***Nephila komaci*** sp. nov. (Fig. 2)

urn:lsid:zoobank.org:act:F31C903E-5D00-4268-A9DB-16BD919F3D16

**Etymology:** Patronym honoring the first author's late friend Andrej Komac.

**Holotype:** Female (nephilid database code ne0729/f1) in NHMW (Naturhistorisches Museum Wien, Vienna, Austria): “Sammlung Reimoser, *Nephila turneri* Blackw., Madagaskar” Collected 31.xii.1938. No precise locality data available.

**Paratype:** Female (ne0140/f1) in PPRI (Plant Protection Research Institute, Pretoria, South Africa; museum code 81/521): “Sodwana Bay, 24.xii.1977–7.i.1978, A. Harrington”, incorrectly identified as “*Nephila inaurata madagascariensis*”. The locality lies at approximately 27°32′S 32°40′E in South Africa, KwaZulu-Natal.

**Other material:** Female (ne2341/f1) in PPRI (2006/1403) from South Africa, KwaZulu-Natal, Tembe Elephant Park, West Muzi Swamp Road (webs between *Acacia* trees), 27°00′S 32°30′E, C. Haddad, 15.vii.2004. Male (ne2342/m1) in PPRI (2007/3262) from South Africa, KwaZulu-Natal, Tembe Elephant Park, near Mahlasela hide (closed woodland/sand), 22°02′47″S 32°26′54″E, C. Haddad, 6.i.2002. Isolated male pedipalp (ne0380/m1) in RMCA (Musée Royal de l'Afrique Centrale, Tervuren, Belgium; 124.867) from Tanzania, Zanzibar (approx. 06°10′S 39°11′E), PLG Benoit, 1.xi.1963.

**Distribution:** South Africa (Maputaland), Tanzania (Zanzibar), Madagascar. Our recent expeditions to Madagascar [27], [28] failed to find *N. komaci* despite focused searches.

**Natural history/ecology:** Mostly unknown, but see above. As with other *Nephila* species, *N. komaci* is predicted to spin a large golden orb web, with a three dimensional barrier web at least in early instars [20]. The two Tembe specimens were collected by beating a large shrub, thus the web was probably 2–4 m above the ground. Two other *Nephila* species (*N. inaurata, N. fenestrata*) are sympatric at Tembe.

**Conservation status:** *Nephila komaci* is evidently rare (37 museum collections were examined in addition to field searches), and may be endangered because its only known habitat, Maputaland coastal forest is increasingly rare [29].

**Diagnosis:** Female *N. komaci* differ from all other African *Nephila* species except *N. sumptuosa* and *N. inaurata* by the shape of the abdomen, which is wide and long, and extends considerably beyond spinnerets (Fig. 2A–B). Female *N. komaci* differ from those of *N. sumptuosa* by the ridged carapace edge (Fig. 2A), the almost unicolorous sternum, and by lacking extensive fields of femoral short macrosetae. They differ from *N. inaurata* by a conspicuous yellow and brown abdominal dorsal pattern (Fig. 2A–B) and the epigynum with slit-like copulatory openings (Fig. 2C–D). The male palp (Fig. 2E–F) differs from all other *Nephila* species by the relatively short embolic conductor (less than 1.5 times cymbium length).

**Description:** *Female* paratype: Total length 39.7. *Prosoma* 14.3 long, 10.9 wide, 8.7 high at head region; dark red-black. High head region, low thoracic region. Carapace densely covered with thin white hairs; mid-carapace humps large and rounded. Carapace lateral edge at thoracic region ridged. Sternum 6.9 long, 5.5 wide, widest anteriorly, with paired sternal humps adjacent to coxae 1–4, the third paired hump enlarged; a large unpaired projection on anterior sternum. Sternum dark red-brown (in alcohol) with a small yellow spot at each paired hump. Labium black, yellow frontally and medially. Maxillae black, medially white. Clypeus height 1.25. Legs and palp unicolor dark red (in alcohol). Leg formula 1, 2, 4, 3. Coxae 3 and 4 with a conspicuous ventral bulge. Femora with sparse warts. Tibiae 1, 2 and 4 with a conspicuous distal tuft of setae. Leg I length 75.4 (femur 21.7, patella 5.1, tibia 18.9, metatarsus 25.4, tarsus 4.3). *Opisthosoma* massive, widest anteriorly, 27.3 long, 12.4 wide (frontally), 12.7 high, extended 4.9 beyond spinnerets. Dorsum (in ethanol) brown with a broad anterior yellow notched pattern, a mid-posterior paired and a caudal unpaired yellow patch; lateral opisthosoma brown with yellow spots and stripes; venter brown, with two irregularly shaped conspicuous yellow transverse bands. *Epigynum* a protruding sclerotized area and a posterior transverse plate with slit-like, medially converging copulatory openings (Fig. 2C–D). Round spermathecae juxtaposed medially. Copulatory ducts complex and long, fertilization ducts massive.

*Male* ne2342 from Tembe, South Africa, compare with Fig. 2: Total length 8.7. *Prosoma* 4.1 long, 2.9 wide, 1.9 high; carapace (in ethanol) light brown in the head region and dark brown in the thoracic region. Sternum 1.84 long, 1.63 wide; yellow-brown, dark gray laterally, with conspicuous paired humps adjacent to coxae 1 and 3, and inconspicuous paired humps adjacent to coxae 2. Eye tapetum in secondary eyes conspicuous and wide. Clypeus height 0.20. Legs yellow-brown, proximal joints dark brown. Both legs 1 missing, leg 2 length 38.5 (femur 8.0, patella 1.7, tibia 6.5, metatarsus 10.7, tarsus 2.7). *Opisthosoma* 5.7 long, 2.0 wide, 1.1 high. Scutum dark brown, with a frontal long paired longitudinal light patch and four posterior small round light patches, lateral opisthosoma black, ventral opisthosoma dark brown-black with a longitudinal paired light band. *Pedipalp* with two distal patellar macrosetae (reconstructed in Fig. 2E), transparent ectal cymbial edge, conspicuous ectal paracymbial setae, and a short, slightly sigmoidal embolic conductor.

**Size variation:** Female prosoma length from 12.3 to 14.3; total length from 32.9 to 39.7 (n = 3). Male variation unknown (n = 1).

**Phylogeny:** The new species belongs to an unnamed African distal *Nephila* clade (Fig. 1B), which justifies its placement in *Nephila*.

## Methods

Taxonomic methods follow recent nephilid treatments [26], [30], [31], all measurements are in millimeters. *Nephila komaci* data added to a nephilid phylogeny [20] produced the same four topologies and preferred hypothesis (Fig. 1C). Although Fig. 1C depicts the evolution of mean female and male size (under squared change parsimony), all statistical tests used log (mean body length  =  average of minimum and maximum values) corrected via independent contrasts [32] using the PDAP module [33] in Mesquite [34]. We construed branch lengths as the count of unambiguous changes plus one (to correct for seven terminal zero length branches). For Monte Carlo simulations in Mesquite, we used an estimate of ancestral body sizes in nephilids (10.0 mm for females, 3.4 for males; linear parsimony reconstruction at the root), as the null hypothesis for body size under no selection. We adjusted the Brownian motion rate parameter so that for each sex the average simulated variance approximated the observed, and simulated body size evolution 15,000 times. SSD is defined as mean female body length: mean male body length. Extreme SSD is defined as SSD value exceeding 5. Using mean prosomal length as a measure of body size, or linear parsimony instead of squared, changes no statistical conclusions.

### Nomenclatural Acts

The electronic version of this document does not represent a published work according to the International Code of Zoological Nomenclature (ICZN), and hence the nomenclatural acts contained in the electronic version are not available under that Code from the electronic edition. Therefore, a separate edition of this document was produced by a method that assures numerous identical and durable copies, and those copies were simultaneously obtainable (from the publication date noted on the first page of this article) for the purpose of providing a public and permanent scientific record, in accordance with Article 8.1 of the Code. The separate print-only edition is available on request from PLoS by sending a request to PLoS ONE, 185 Berry Street, Suite 3100, San Francisco, CA 94107, USA along with a check for $10 (to cover printing and postage) payable to “Public Library of Science”.

The online version of the article is archived and available from the following digital repositories: PubMedCentral (www.pubmedcentral.nih.gov/), LOCKSS (http://www.lockss.org/lockss/), Smithsonian Institution (http://hdl.handle.net/10088/8183), and Nephilidae.com: A web resource for nephilid spiders (Araneae, Araneoidea, Nephilidae) (http://www.nephilidae.com). In addition, this published work and the nomenclatural acts it contains have been registered in ZooBank, the proposed online registration system for the ICZN. The ZooBank LSIDs (Life Science Identifiers) can be resolved and the associated information viewed through any standard web browser by appending the LSID to the prefix “http://zoobank.org/”. The LSID for this publication is: urn:lsid:zoobank.org:pub:AB864145-ED15-403D-BADA-C617E322ED4B.
